# Mediterranean Journal of Rheumatology September 2019 Issue Highlights

**DOI:** 10.31138/mjr.30.3.139

**Published:** 2019-09-30

**Authors:** Lazaros I. Sakkas

**Affiliations:** Department of Rheumatology and Clinical Immunology, Faculty of Medicine, School of Health Sciences, University of Thessaly, Larissa, Greece

The September issue of the MJR offers readers interesting reviews and case reports.

Versini et al.^6^ examined the development of ischemic heart disease in 155 patients with Takayasu arteritis (TA) using the Clalit Health Services, the largest Health Maintenance Organization in Israel. Ischemic heart disease was increased in TA relative to age- and gender-matched controls (32.3% vs 8.9%). Also, the 15-year survival was reduced in TA with mortality 21.9% vs 8.8% in controls (HR:2.58).

Markatseli et al.^7^ examined the efficacy of tocilizumab in patients with rheumatoid arthritis in a 6-month single-arm study of routine clinical practice in Greece. One hundred and eighty-three patients received tocilizumab IV infusion every 4 weeks. At 6 months, DAS28(ESR) changed by –1.3, and CDAI dropped from 29.6 to 16.7. Good-moderate response was achieved by 89.1% of patients and remission by 23.5% of patients. There were no unexpected adverse effects.

Kelly’s excellent review is focused on lung disease, and particularly on interstitial lung disease (ILD) in rheumatic disorders.^2^ Pleural effusion, most common in rheumatoid arthritis (RA) and systemic lupus erythematosus (SLE) have become relatively rare in recent years. Pulmonary hypertension, common in systemic sclerosis (SSc), occurs in SLE patients with antiphospholipid antibodies. ILD is very common in SSc, where it also causes pulmonary hypertension, but also in RA. Kelly provided data from the BRILL network for RA-ILD. Patients on rituximab had better survival than patients on anti-TNFα therapy, and suggests that patients with RA-ILD who are seropositive should be treated with rituximab, while seronegative patients should be treated with abatacept or tocilizumab. Sidiropoulos et al.^1^ discuss the evolution of rheumatoid arthritis (RA) treatment in Greece over 20 years since the arrival of biologic agents. The Hellenic Society for Rheumatology was quick to adapt EULAR recommendations on treatment and update them frequently. Also, early on the Hellenic Registry of Biologic Therapies was established which, by 2015, included 2,874 patients (1,608 patients with RA) from eight academic centers. The introduction of the e-prescription system, and a new country-wide database for RA mainly from tertiary referral centers in Greece, revealed that 42% of RA patients are on biologics.

In an excellent paper, Mende et al.^3^ review autoantibodies in myositis and group them into myositis-specific autoantibodies (MSA) and myositis-associated autoantibodies. MSA are increasingly used as biomarkers correlated with clinical phenotypes and offer prognostic information. For instance, autoantibodies against SAE1, TIF1-γ, NXP2 are associated with cancer. In this review, readers will find many useful tips for their practice.

Ceribelli et al.^4^ in an excellent paper pinpoint differences between males and females in innate and adaptive immunity and examine the link of genes and environmental factors with rheumatic diseases in females.

Case reports of interesting and/or unusual cases enrich readers with practical knowledge. Kourkouni et al.^8^ report on patient with fever and severe interstitial lung disease (ILD) requiring oxygen supplementation. The patient did not respond to antibiotics and finally she was diagnosed with antisynthetase syndrome, treated with steroids and rituximab and had a good recovery.

Grigoropoulos et al.^5^ report on a patient on tofacitinib, an oral JAK inhibitor, who developed *P. jirovecii* pneumonia, an uncommon infection in tofacitinib-treated patients.

Bounia et al.^9^ report on a patient with fever, oligoarthritis, and bullous skin lesions which evolved into pyoderma gangrenosum-like lesions. The patient, although denied any intestinal manifestation, underwent colonoscopy which established the diagnosis of Crohn’s disease, and received infliximab with resolution of skin lesions.

## References

[1] Sidiropoulos P Sfikakis PP Boumpas D Vassilopoulos D Twenty Years of Targeted Treatment in Rheumatoid Arthritis in the Greek Databases: Achievements and Unmet Needs. Mediterr J Rheumatol 2019;30(3):141–6 [10.31138/mjr.30.3.141]. PMC7045862 32185356

[2] Kelly C Lung Disease in Rheumatic Disorders. Mediterr J Rheumatol 2019;30(3):147–54 [10.31138/mjr.30.3.147]. PMC7045857 32185357

[3] Mende M Borchardt-Lohölter V Meyer W Scheper T Schlumberger W Autoantibodies in Myositis - How to Achieve a Comprehensive Strategy for Serological Testing. Mediterr J Rheumatol 2019;30(3):155–61 [10.31138/mjr.30.3.155]. PMC7045863 32185358

[4] Ceribelli A De Santis M Selmi C Sex and autoimmune disease: Four mechanisms pointing at women. Mediterr J Rheumatol 2019;30(3):162–6 [10.31138/mjr.30.3.162]. PMC7045856 32185359

[5] Grigoropoulos I Thomas K Christoforou P Fanidi I Papavdi M Kyriakou F Deutsch M Pirounaki M Vassilopoulos D *Pneumocystis Jirovecii* Pneumonia after Initiation of Tofacitinib Therapy in Rheumatoid Arthritis: Case-Based Review. Mediterr J Rheumatol 2019;30(3):167–70 [10.31138/mjr.30.3.167]. PMC7045858 32185360

[6] Versini M Tiosano S Sharif K Mahroum N Watad A Comaneshter D Shalom G Shoenfeld Y Cohen AD Amital H Association between Takayasu arteritis and ischemic heart disease: a cohort study. Mediterr J Rheumatol 2019;30(3):171–6 [10.31138/mjr.30.3.171]. PMC7045855 32185361

[7] Markatseli TE Theodoridou A Zakalka M Koukli E Triantafyllidou E Tsalavos S Andrianakos A Drosos AA Persistence and Adherence during the First Six Months of Tocilizumab Treatment Among Rheumatoid Arthritis Patients in Routine Clinical Practice in Greece. Results from the Single Arm REMISSION II Study (NCT01649817). Mediterr J Rheumatol 2019;30(3):177–85 [10.31138/mjr.30.3.177]. PMC7045860 32185362

[8] Kourkouni E Mitsogiannis G Simopoulou T Liaskos C Katsiari CG Daniil Z Gourgoulianis K Bogdanos DP Sakkas LI Interstitial lung disease in anti-synthetase syndrome. Mediterr J Rheumatol 2019;30(3):186–9 [10.31138/mjr.30.3.186]. PMC7045864 32185363

[9] Bounia KA Konstantopoulou GM Liossis SNC A 40-Year-Old Woman with Asymmetric Arthritis and Skin Lesions. Mediterr J Rheumatol 2019;30(3):190–3 [10.31138/mjr.30.3.190]. PMC7045859 32185364

